# Novel Osmoprotective DOPC-DMPC Liposomes Loaded with Antihypertensive Drugs as Potential Strategy for Glaucoma Treatment

**DOI:** 10.3390/pharmaceutics14071405

**Published:** 2022-07-04

**Authors:** Miriam Ana González-Cela-Casamayor, José Javier López-Cano, Irene Bravo-Osuna, Vanessa Andrés-Guerrero, Marta Vicario-de-la-Torre, Manuel Guzmán-Navarro, José Manuel Benítez-del-Castillo, Rocío Herrero-Vanrell, Irene Teresa Molina-Martínez

**Affiliations:** 1 Innovation, Therapy and Pharmaceutical Development in Ophthalmology (InnOftal) Research Group, Universidad Complutense de Madrid (UCM), 28040 Madrid, Spain; mirigo04@ucm.es (M.A.G.-C.-C.); josejavl@ucm.es (J.J.L.-C.); ibravo@ucm.es (I.B.-O.); vandres@ucm.es (V.A.-G.); mvicario@ucm.es (M.V.-d.-l.-T.); jmbenitezcastillo@med.ucm.es (J.M.B.-d.-C.); 2 Department of Pharmaceutics and Food Technology, Facultad de Farmacia, Universidad Complutense de Madrid (UCM), IdISSC, 28040 Madrid, Spain; 3 University Institute of Industrial Pharmacy (IUFI), Facultad de Farmacia, Universidad Complutense de Madrid, 28040 Madrid, Spain; 4 Biomedical Sciences Department, Pharmacy and Pharmaceutical Technology Unit, Facultad de Farmacia, Universidad de Alcalá, 28801 Madrid, Spain; manuel.guzman@uah.es; 5 Ocular Surface and Inflammation Unit (USIO), Departamento de Inmunología, Oftalmología y OLR, Hospital Clínico San Carlos, Universidad Complutense de Madrid (UCM), IdISSC, 28040 Madrid, Spain

**Keywords:** hyperosmolarity, glaucoma, liposomes, DED, hypotensive, synthetic phospholipids

## Abstract

Glaucoma is a group of chronic irreversible neuropathies that affect the retina and the optic nerve. It is considered one of the leading causes of blindness in the world. Although it can be due to various causes, the most important modifiable risk factor is the elevated intraocular pressure (IOP). In this case, the treatment of choice consists of instilling antihypertensive formulations on the ocular surface. The chronicity of the pathology, together with the low bioavailability of the drugs that are applied on the ocular surface, make it necessary to instill the formulations very frequently, which is associated, in many cases, with the appearance of dry eye disease (DED). The objective of this work is the design of topical ocular formulations capable of treating glaucoma and, at the same time, preventing DED. For this, two liposome formulations, loaded with brimonidine or with travoprost, were Tadeveloped using synthetic phospholipids and enriched by the addition of compounds with osmoprotective activity. The proposed formulations not only presented physicochemical characteristics (size, pH, osmolarity, surface tension, and viscosity) and encapsulation efficiency values (EE% of 24.78% and ≥99.01% for brimonidine and travoprost, respectively) suitable for ocular surface administration, but also showed good tolerance in human corneal and conjunctival cell cultures, as well as an in vitro osmoprotective activity. The hypotensive effect of both liposomal formulations was evaluated in normotensive albino New Zealand rabbits, showing a faster and longer lasting reduction of intraocular pressure in comparison to the corresponding commercialized products used as control. According to these results, the hypotensive liposomal formulations combined with osmoprotective agents would result in a very promising platform for the treatment of glaucoma and the simultaneous protection of the ocular surface.

## 1. Introduction

Although there are several types of glaucoma, all of them are characterized by the progressive death of the retinal ganglion cells (RGC) whose axons form the optic nerve. Due to the special arrangement of these axons in the optic nerve head, the patient begins to lose peripheral vision, gradually turning into tube-shaped vision and finally leading to total blindness. Glaucoma is one of the main causes of blindness in the world and it is estimated that in 2040 there will be more than 111.8 million affected [1,2]. One of the main modifiable risk factors in glaucoma is the increase in intraocular pressure (IOP), the use of topical antihypertensive drugs being the first line of treatment in those cases [3]. High intraocular pressure compromises blood flow and produces a damage of the optic nerve and retina [4]. As reported by Philip C Maier in a meta-analysis, reducing IOP in glaucoma patients decreased long-term vision loss [5]. In therapeutics, there are many ocular antihypertensive drugs available in chronic topical treatments, such as the α2-adrenergic receptor agonist brimonidine [6] and prostaglandin PGF2 α analogue travoprost [7].

Ocular topical administration is the most employed route to control IOP in glaucoma. Unfortunately, there are several problems that limit the effectiveness of treatments and follow-up by patients: (I) **low ocular bioavailability** [8] and (II) **development of dry eye disease (DED)** [6].

Several technological strategies have been proposed to extend the ocular residence time of drugs, and hence **to increase their ocular bioavailability** [9]. In this sense, the use of nanotechnology allows bioavailability to increase and also protects the drug from degradation [10]. Furthermore, due to their small size, they can establish intimate interactions with biological tissues. Among the different nanosystems currently available for this purpose, liposomes, composed by an aqueous core surrounded by one or several lipid bilayers [10], were the first to appear, and one of the most promising. They offer high biocompatibility and biodegradability [11] and are very versatile, so they can entrap hydrophilic drugs in the aqueous core and can also incorporate lipophilic drugs in the lipid bilayers [4].

In the case of ocular topical administration, previous studies on corneal cells have shown that liposomes are able to penetrate into the cells of the ocular surface [12]. Furthermore, the lipid bilayer of liposomes is mainly composed of phospholipids, widely presented in the tear film composition. Consequently, liposomes are able to supplement the lipid layer of the tear film and prevent tear evaporation, improving the symptoms of DED [9,13]. In recent years, the use of synthetic phospholipids has increased because of their advantages in terms of standardization, characterization, and potential scaling of liposome production [14,15].

The addition of mucoadhesive polymers in eye-drops have been also proposed as a tool to increase the residence time of ocular topical formulation. In addition, the viscosity generally provided by these polymers in solution allows the formation of a layer that covers the ocular surface [16,17]. Studies carried out with liposomal formulations containing HPMC (hydroxypropyl methylcellulose) have shown an improvement on the loaded drug efficacy after ocular instillation [4]. In addition, previous studies carried out in our research group showed that the inclusion of HPMC in the eye-drops has a beneficial effect on their tolerance on the ocular surface [18].

The precorneal film is composed of an external lipid layer (phospholipids, cholesterol fatty acids, etc.) in contact with the air. Behind this lipid layer there is an aqueous layer where there are electrolytes, mucin, proteins, among others, that are in close contact with the conjunctival and corneal epithelium [19]. **Dry eye disease (DED)** is caused by a disruption of the homeostasis of this tear film produced by an increase in tear evaporation (evaporative dry eye) or a deficient tear production (aqueous deficient dry eye). This promotes the permanent contact of conjunctiva and cornea cells with a hyperosmolar aqueous media [20] which subsequently produces damage and inflammation (increased matrix metalloproteinases and activation of proinflammatory cytokine cascades) on these tissues [20,21,22]. All these events generate apoptosis of ocular surface cells, which, in turn, destabilizes the tear film even more, creating a vicious cycle [21]. This is the reason why the use of osmoprotectants such as ribitol [23] and taurine [24] has emerged as a therapeutic strategy to protect the ocular surface [25]. Furthermore, authors have linked oxidative stress to the production of damage and inflammation in the conjunctiva of these patients [26]. For this reason, the inclusion of antioxidants in the lipid bilayer, such as vitamin E or ubiquinol [27], would result in being advantageous to ameliorate DED symptoms.

In addition, recent studies link topical antiglaucomatous treatments with the dysfunction of the meibomian glands, responsible for producing precorneal film mucins [28,29], which can also induce DED [28]. In most cases, excipients of the formulations, mainly preservatives, are responsible for the ocular surface damage observed after chronic administrations. This effect has been described mostly for benzalkonium chloride (BAK), one of the most commonly used preservatives in topical formulations [30,31]. However, the hypotensive substances could also produce a damage in the ocular surface [31]. In fact, recent studies have shown an increase of osmolarity, tear film instability, and conjunctival hyperemia in patients with chronic treatments of some topical antiglaucomatous agents, as it is the case of timolol maleate or prostaglandin analogues [29]. 

As the objective of this work is the design of topical ocular formulations capable of treating glaucoma and, while at the same time preventing DED, several strategies have been combined for this purpose. In order to increase their ocular bioavailability, brimonidine and travoprost have been formulated in liposomes to promote an intimate contact of the formulation with the ocular surface and also because the presence of lipids on its composition would help to preserve the integrity of the lipid external layer of the tear film, preventing the aqueous evaporation. Synthetic phospholypids have been used to elaborate the liposomes. Furthermore, vitamin E and ubiquinol were incorporated in the lipid bilayer as antioxidants. In addition, osmoprotective components (taurine and ribitol) have been included in the aqueous media. Finally, formulations were prepared with and without HPMC in the external aqueous media to evaluate its potential positive effect on the formulation retention time and tolerance on the ocular surface. The physicochemical characteristics of the liposomes have been optimized for ocular instillation. The in vitro tolerance of the different formulations was evaluated in human corneal and conjunctival cells. Additionally, the osmoprotective activity was studied in an in vitro model of hyperosmolar stress. Finally, the in vivo hypotensive effect of the systems proposed was measured in normotensive New Zealand albino rabbits.

## 2. Materials and Methods

### 2.1. Materials

Synthetic phospholipids 1,2-dioleoyl-sn-glycero-3-phosphocholine (DOPC) and 1,2-dimyristoyl-sn-glycero-3-phosphocholine (DMPM) were purchased from Lipoid GmbH (Ludwigshafen, Germany). The other lipid components of liposomes, Cholesterol (≥99%), α-Tocopherol acetate (≥96%), and Ubiquinol (USP reference standard), were supplied by Sigma Aldrich, as well as the aqueous compounds Ribitol (Adonitol, ≥99%), Taurine (≥99%), Sodium tetraborate (Na_2_B_4_O_7_, ≥99.5%), and Boric Acid (H_3_BO_3_, ≥99.5%). Brimonidine was obtained in Fagron Iberica (Terrassa, Spain) and travoprost in MedChemExpress (Monmouth Junction, NJ, USA).

Defined Trypsin Inhibitor was supplied by Life Technologies (Madrid, Spain). Fetal Bovine Serum was supplied by Thermo Fisher (Madrid, Spain). Trypan-Blue, MTT [3-(4,5-dimethylthiazol-2-yl)-2,5-diphenyltetrazolium bromide], Dimethyl sulfoxide (DMSO), Dulbecco’s phosphate-buffered saline (DPBS), Trypsin-EDTA 0.05%, Sodium Chloride (NaCl), Sodium Chloride 5M, and 2% Gelatin solution were provided by Sigma Aldrich (Madrid, Spain). T-75 flasks and polystyrene 15 mL tubes were from Sarstedt (Madrid, Spain).

### 2.2. Liposome Preparation

Two liposomal formulations containing Brimonidine (FL-B) or Travoprost (FL-T) (Table 1) were prepared using the lipid film hydration method described by Bangham, including modifications [32]. The phospholipid composition of the lipid bilayer was a mixture of 7.5 mg/mL DOPC and 2.5 mg/mL DMPC, obtained a final 10 mg/mL concentration of phospholipids. In addition, cholesterol, and the antioxidants vitamin E and ubiquinol were included. The weight ratio of the lipid bilayer (DOPC:DMPC:Ch:ViE:Ubiq) was 6:2:1:0.08:0.02. As previously highlighted, phosphatidylcholine derivatives supplement the tear film. 

The aqueous dispersion was composed by the osmoprotectants Ribitol 0.5% and Taurine 0.5% and a borate buffer (H_3_BO_3_ 8.38% and Na_2_B_4_O_7_ 0.755%) to maintain the pH. The final formulation was tested with and without the polymer hydroxypropylmethylcellulose (HPMC) 0.2%.

All the components of the lipid bilayer were dissolved in chloroform and reduced pressure was applied in a rotary evaporator (Buchi R-205, Massó Analítica S.A., España), first at 100 mPa for 30 min, and subsequently at 50 mPa for another 30 min, which obtained a layer covering the round-bottomed flask. The temperature was maintained at 32 °C. To remove the remained chloroform, a flux of nitrogen was employed. The aqueous dispersion glass beads were added to the layer and the formulation remained at room temperature, protected by the light for 2 h (maturation). Subsequently, the liposomal formulation was sonicated during 15 min in an ultrasound bath (Bandelin^®^ Sonorex Digiplus, DL 510 H, Berlin, Germany). Finally, the liposomal formulations were extruded with 0.8 and 0.2 μm filters to get homogeneous sizes. 

All formulations were prepared at double concentration, so the final step was to make a 1:1 dilution using the aqueous phase (FL-B and FL-T) or the aqueous phase with 0.4% HPMC (FLP-B and FLP-T).

The active ingredients for each type of formulation were added during preparation. For formulations containing the hydrophilic active ingredient brimonidine (FL-B and FLP-B), brimonidine was added in the aqueous dispersion to obtain a final concentration of 2 mg/mL. In the case of the formulations containing the hydrophobic active substance travoprost (FL-T and FLP-T), it was dissolved in chloroform with the rest of the lipid bilayer components, giving a final concentration of 40 µg/mL.

### 2.3. Determination of the Physicochemical Properties of the Liposomal Formulations

The technique used to measure the size and the size distribution profile of the liposomal formulation was the dynamic light scattering. The equipment used was the Microtrac^®^ S3500 Series (Montgomeryville, PA, USA). The polydispersion index (PDI) was also calculated. For the evaluation of the zeta potential of the formulations, the Autosizer 4700 (Malvern, UK) was used.

Osmolarity measurements were acquired by a vapor osmometer (Fiske Micro-Osmometer, model 210). The calibration was performed with the 50, 290, and 850 mOsm/L standards. 

The pH measurement was determined using the pH-meter (model GLP-2, CRISON). The calibration was performed with pH 4 and pH 7 standards. 

The surface tension was analyzed with the Wilhelmy plate method and calibrated with water (68–72 mN/m) using the tensiometer K-11 (Kruss GmbH, Hamburg, Germany). The temperature of the samples was the one corresponding to ocular surface temperature (32 °C). 

The Discovery HR-1 hybrid Rheometer (New Castle, DE, USA) with a parallel plate (69 mm) was employed to perform viscosity. The study was conducted in 20 steps, with a shear rate increasing from 0 to 1000 s^−1^.

### 2.4. HPLC Quantification and Encapsulation Efficiency of the Active Ingredients

The quantification of active substances (brimonidine and travoprost) was carried out by high-performance liquid chromatography (HPLC) using the isocratic method. The equipment used to carry out the experiments is the RP-HPLC Acquity Arc Bio^®^ (Waters, Madrid, Spain). The RP-HPLC was equipped with a photodiode array detector (2998 PDA Detector), a bioSample Manager FTN-R, and a bioQuaternary Solvent manager-R. 

The quantification of brimonidine was performed using a previously described method with some modifications [33]. Tracer excell 120 ODSA 5 µm 15 × 0.4 TR-015694 column was employed (Teknocroma). The column was kept at a temperature of 30 °C, the flow rate was 1 mL/min, the injection volume was 10 µL, and a detection wavelength of 246.1 nm was used. The calibration curve was performed using a 500 µg/mL brimonidine standard. The different curve concentrations (40, 20, 15, 10, 5, and 1.5 µg/mL) were prepared in methanol. The solution used as the mobile phase was composed by phosphate buffer (KH_2_PO_4_ 10mM acidified with phosphoric acid to obtain a 3.5 pH) with TEA 0.5% in water and Methanol (85:15). The liposomes were freeze-dried after preparation. Subsequently they were dissolved in methanol, centrifuged and filtered, and the yield was calculated (1). The percentage of drug lost during the manufacturing process was calculated as the difference between the yield obtained and 100%.

(1) $$\mathrm{Yield}\%=\frac{Real\;amount\;of\;Brimonidine\;in\;the\;formulation}{Theoretical\;amount\;of\;brimonidine\;in\;the\;formulation}\times 100$$

To quantify the encapsulation efficiency (EE) of brimonidine, the FL-B liposomal formulation was subjected to ultrafiltration to separate the liposomes from the aqueous dispersion, and then the free fraction of brimonidine in the supernatant was determined by HPLC analysis. To determine the free concentration of brimonidine present in the formulation, liposomes were subjected to ultrafiltration using 0.5 mL tubes with centrifugal filters of 50 kD (Ultracel^®^). The liposomes were diluted 1:10, and 0.25 mL of the solution was added to the ultrafiltration tubes. Subsequently, they were centrifuged at 14,000 rpm for 5 min. Finally, they were diluted with methanol (1:10) and quantified by HPLC.

The concentration of brimonidine encapsulated in the liposomes was indirectly calculated from the amount of total brimonidine in the liposomal formulation and the fraction of free brimonidine in the aqueous phase (2).

(2) $$\mathrm{EE}\%=\frac{\left(Total\;amount\;of\;Brimonidine-Free\;amount\;of\;Brimonidine\right)}{Total\;amount\;of\;Brimonidine}\times 100$$

The quantitative analysis of travoprost was based on the method described in the United States Pharmacopeia (USP-NF 2021) with some modifications [34]. Ascentis C18 5 µm 25 × 0.46 cm column was used. The column was kept at 30 °C, and a flow rate of 1 mL/min and an injection volume of 10 µL were used. The maximal absorption quantification was fixed 222.5 nm wavelength. Different curve points in ethanol absolute (50, 25, 10, 5, 2.5, 1, 0.5, and 0.25 µg/mL) were prepared from a calibration curve using a 1 mg/mL travoprost standard. The solution used as mobile phase consisted of water acidified with 0.1% TFA and acetonitrile HPLC grade (40:60). The liposomes were freeze-dried, dissolved in absolute ethanol, centrifuged, and filtered.

The EE was calculated as previously explained for brimonidine, with the peculiarity that the limit of detection and limit of quantification had to be calculated to estimate the minimum EE (%). The free concentration was also determined using the centrifugal filters of 50 kDa (14,000 rpm for 5 min), using a volume of 0.5 mL. As travoprost was not detected in the aqueous dispersion, it was necessary to determine the Y-intercept, slope, and the limit of quantification to determine the minimum encapsulation efficiency [35,36].

### 2.5. In Vitro Studies in Human Corneal Cell Lines

#### 2.5.1. Cell Cultures

The cytotoxicity and osmoprotection assays were conducted with immortalized human corneal epithelial cell line (hTERT-HCECs) (Evercyte GmbH, Vienna, Austria). Corneal cells were maintained with EpiLife^®^ medium supplemented with EDGS^®^ and 1% penicillin-streptomycin. The medium was changed every 2–3 days and the experiments were carried out with 5–15 passages. T-75 culture flasks were selected for the subcultures (80–85% confluence), previously coated with 2% gelatin solution. Cells were washed with DPBS, and subsequently 0.005% trypsin-EDTA was used to detach the cells from the flask. The trypsinisation time was 5 min, followed by light tapping on the flask. Once peeled off, trypsin inhibitor was used to neutralize trypsin and the cells were centrifuged (5430R Centrifuge, Eppendorf, Madrid, Spain) at 850× *g* in a 15 mL tube to get a pellet. The cell pellet was resuspended and added to new flasks with culture medium. 

For the cytotoxicity assays, an immortalized human conjunctival epithelial cell line (IM-HConEpiC) (Innoprot, Bizkaia, Spain) was also used. The medium was renewed every two days, and its maintenance was carried out in collagen coated flasks using the Collagen I Coating Kit (1 mg/mL) (Innoprot, Bizkaia, Spain) and the IM-Ocular Epithelial Cell Medium Kit (Innoprot, Bizkaia, Spain). Subcultures were performed similarly to the corneal cell line. The passages used to assay performance ranged between 2–10.

Cell cultures were maintained in a saturated humidified atmosphere at 37 °C and 5% CO_2_.

#### 2.5.2. In Vitro Tolerance Determination in Human Corneal hTERT-HCECs Cells

The cytotoxicity of the different liposomal formulations containing 2 mg/mL brimonidine (FLB and FLP-B) or 40 µg/mL travoprost (FLT and FLPT) was determined. In addition, two commercial formulations containing brimonidine (CCB) and travoprost (CCT) were used for comparison. The evaluation was carried out by the 3-(4,5-dimethylthiazol-2-yl)-2,5-diphenyltetrazolium bromide (MTT) assay, described before by our group [36]. Cell cultures were allowed to grow until 80–85% confluency and then cultured in 96-well plates at 20,000 cells/well. 

After seeding and overnight incubation (16 h), the cells were exposed to the formulations, using a volume of 100 µL/well of formulation and 100 µL/well of the previously described culture medium. To simulate acute exposure, the human corneal cells were exposed for 1 h to formulations. On the other hand, exposure was also performed for 4 h to simulate a chronic treatment [32].

For cell viability determination, the supernatant was discarded and a solution of 0.3% MTT in culture medium was added. MTT solution was added at a volume of 100 µL/well. The exposure lasted 4 h, the time necessary for live cells to oxidize MTT to its formazan salt. To dissolve the formazan crystals formed, the reagent DMSO was used. The supernatant was first discarded and then 100 µL/well of DMSO was added. The absorbances were read in a spectrophotometer, using a wavelength of 550 nm, with prior shaking of the plate for 5 min.

The positive control selected was 0.005% BAK, a preservative commonly included in topical ophthalmic formulations, causing poor tolerance and cell dead. BAK was selected as a positive control due to findings by other authors, which links it to the development of DED and inflammatory processes in corneal and conjunctival cells [31,37]. 0.9% NaCl was used as a negative control, equated to 100% cell viability.

#### 2.5.3. In Vitro Tolerance Determination in Human Conjunctival IM-HConEpiC Cells

Toxicity in vitro assays with an immortalized human conjunctival epithelial cell line (IM-HConEpiC) were performed in a similar way to the previous section. In this case, the density of cells seeded was 25,000 cells/well. Cells were exposed to the final 2 mg/mL brimonidine and 40 µg/mL travoprost containing formulations (FLB, FLT, FLP-B, and FLP-T) during 1 and 4 h. Cytotoxicity was measured with the MTT technique in the same way as in the previous section. As negative and positive control, 0.9% NaCl and 0.005% BAK, respectively, were also used.

#### 2.5.4. Osmoprotection Studies by Hyperosmolar Stress Simulation in hTERT-HCECs Corneal Cells

Osmoprotection was performed in a previously developed hypertonic stress model in hTERT-HCECs cells [38]. This model allows the screening of osmoprotective substances or topical ophthalmic formulations in a hypertonic media. Briefly, 20,000 cells/well were seeded and incubated overnight in 96-well plates. Subsequently, the supernatants were aspirated, and the cells exposed to the different developed formulations for 4 h (100 µL of medium and 100 µL of formulation in each well) to simulate a preventive treatment to cope with the onset of DED. 4 h pre-treatment with 0.9% NaCl was used as positive control.

After that, supernatants were removed, and the previously treated cells were exposed to hyperosmolar conditions 470 mOsm/L for 16 h, except for the negative control, which was exposed to isotonic conditions (NaCl 0.9%). Finally, the supernatants were discarded and 100 µL/well of 0.33 mg/mL MTT solution was added to determine cell viability, as described in the previous section.

### 2.6. In Vivo Studies

#### 2.6.1. Animals

New Zealand albino rabbits (San Bernardo Farm, Spain) with approximately 3.5 kg weight were used to carry out the in vivo studies in accordance with the relevant regulations on animal experimentation (European Communities Council Directive (86/609/EEC) the Statement for the Use of Animals in Ophthalmic Vision Research in Association for Research in Vision and Ophthalmology (ARVO) [39] and Spanish Regulation of Experimental Studies with Animals (RD 53/2013 1 February modified by the RD 118/2021 23 February). The protocol code was: PROEX 114.4/21 (16 July 2020).

The animals were kept under inverted light cycle conditions and at a temperature of 22 °C and 50% humidity in a controlled atmosphere.

#### 2.6.2. In Vivo Hypotensive Studies

The in vivo hypotensive effect was carried out with an instillation of 25 µL of each formulation into both eyes at 5 male New Zealand albino rabbits (10 eyes). For rabbit recovery, animals were maintained with no treatment for at least 48 h. IOP measurements were performed using a tonometer (Tonovet) and tonometer probes (Tiolat). To establish 100% intraocular pressure (Baseline), measurements were taken half an hour before (t = −0.5) and at the time of administration (t = 0). Subsequently, intraocular pressure measurements were taken hourly for an interval of 11 h and then at 24 h. In cases in which IOP pressure remained below 100% after 24 h, measurements were taken at 28, 32, and up to 48 h.

The decrease in IOP achieved by the liposomal formulations was firstly compared to those obtained with the base vehicle (FL and FLP without active ingredient). Subsequently, the hypotension achieved by a commercial formulation containing brimonidine (Alphagan^®^) (CCB) was compared with 2 mg/mL brimonidine liposomal formulations with and without polymer (FL-B and FLP-B). For the comparison of the FL-T and FLP-T (40 µg/mL) formulations, a commercial formulation with travoprost (Travatan^®^) (CCT) was used.

To analyze the results, we established the time at which the IOP was significantly reduced (*p* < 0.05) from baseline (Onset time) for each formulation and until what time this difference was significant (effective time). The times for calculating the areas under the curve (AUC_0–t_) were selected consequently. Data treatment was applied calculating the AUC_0–t_ and maximal IOP reduction (ΔIOP_max_) produced by each formulation and testing its significance. In order to study whether one formulation had an advantage over another, it was studied whether the 95 CI interval of the difference between them did not include 0 (Relationship between the two mean difference test (*p*-value of the Student’s *t*-test)).

### 2.7. Data Analysis

The software used to carry out the statistical analysis of in vitro studies was the GraphPad software Inc. Prism Version 8.0.2, US. For all data, measurements were taken in triplicate (mean ± SD). Statistical significance was established using the Ordinary one-way ANOVA test using the Sidak’s multiple comparisons test (α ≤ 0.05).

Statistical treatment for IOP curves and areas was performed with Statgraphics 19, using description and comparison by hypothesis testing. It was considered significant at *p* value < 0.05%.

## 3. Results

### 3.1. Physicochemical Determination

**Figure 1:**
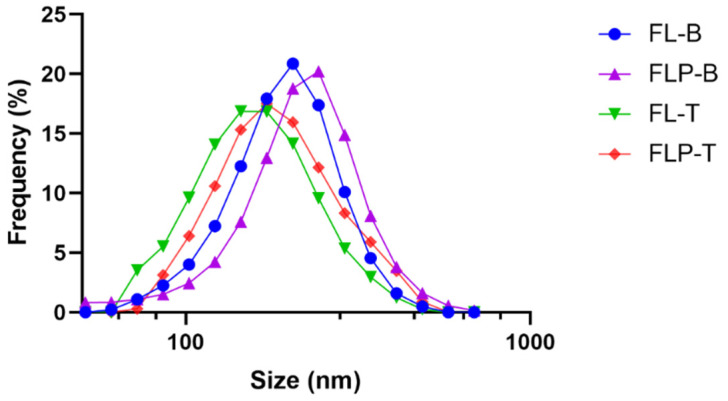
Particle size distribution of liposomal formulation containing brimonidine with (FLP-B) and without (FL-B) HPMC 0.2% and travoprost with (FLP-T) and without (FL-T) HPMC 0.2% represented in a semi-logarithmic scale.

The different liposomal formulations containing brimonidine or travoprost were characterized in terms of size, pH, osmolarity, surface tension, and viscosity (Table 2). The sizes of the liposomal vesicles were around 200 nm with a unimodal distribution (Figure 1). The average particle size resulted in values between 150 and 220 nm, increasing in both formulations when HPMC was included in the aqueous dispersion and maintaining the unimodal size distribution. The polydispersion index was, in all cases, between 0.10 and 0.16. The zeta potential of all formulations was found to be between −10 and 10 mV, which means a neutral zeta potential.

The pH of all formulations was neutral, being in the 6.5–7 range. All formulations were hypotonic, with values between 220 and 230 mOsm/L. 

For the determination of the adequate extensibility of the formulations, surface tension and viscosity were measured. The surface tension values of all formulations were in the range of 25–30 mN/m. The viscosity of the liposomal formulations of both active ingredients was close to 1 mPa·s, while it increased when the polymer was included in both formulations.

### 3.2. HPLC Quantification and Encapsulation Efficiency of Active Ingredients

The active ingredients brimonidine and travoprost included in the liposomal formulations were determined in terms of yield and encapsulation efficiency (Data shown in Table 3).

Focusing on the liposomal formulation containing brimonidine (FL-B), the yield was around 100%, meaning that all drug initially included during the elaboration was retained in the formulation. However, according to %EE values, only about 25% of the drug was retained inside the liposomes, while the remaining 75% of brimonidine was found in the aqueous dispersion. In contrast, the yield data of the formulation containing travoprost (FL-T) suggested that around 11% of the drug initially used for the preparation of the liposomes was lost throughout the elaboration process. However, an encapsulation efficiency of this liposoluble active ingredient of values close to 100% were obtained due to the fact that travoprost was not detected in the aqueous dispersion by HPLC. The slope was 11,446 and the Y-intercept was −89.49 (Standard error: 397.1). The resulted Limit of detection (LD) and Limit of quantification (LQ) were 0.114 µg/mL and 0.347 µg/mL, respectively. 

### 3.3. In Vitro Studies

#### 3.3.1. In Vitro Tolerance Evaluation in hTERT-HCECs

**Figure 2:**
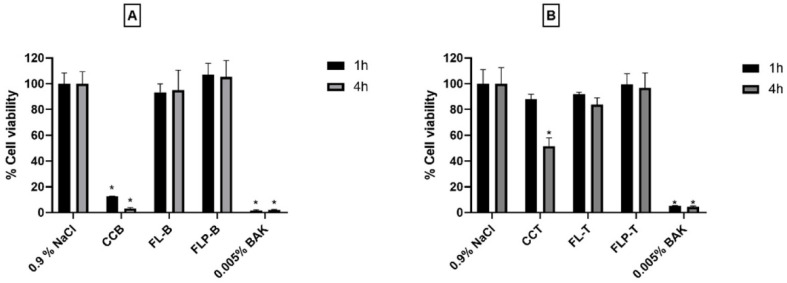
In vitro toxicity evaluation in human corneal hTERT-HCECs cells of liposomal formulations containing brimonidine (FL-B and FLP-B) (**A**) or travoprost (FL-T and FLP-T) (**B**) after 1 and 4 h exposure. FL-B: Brimonidine 0.2%, DOPC 7.5 mg/mL, DMPC 2.5 mg/mL, vitamin E 0.1 mg/mL Ubiquinol 0.025 mg/mL, Taurine 0.5%, Ribitol 0.5%; FLP-B: Brimonidine 0.2%, DOPC 7.5 mg/mL, DMPC 2.5 mg/mL, Vitamin E 0.1 mg/mL Ubiquinol 0.025 mg/mL, Taurine 0.5%, Ribitol 0.5%, HPMC 0.2%. FL-T: 40 µg/mL, DOPC 7.5 mg/mL, DMPC 2.5 mg/mL, vitamin E 0.1 mg/mL Ubiquinol 0.025 mg/mL, Taurine 0.5%, Ribitol 0.5%; FLP-T: Travoprost 40 µg/mL, DOPC 7.5 mg/mL, DMPC 2.5 mg/mL, vitamin E 0.1 mg/mL Ubiquinol 0.025 mg/mL, Taurine 0.5%, Ribitol 0.5%, HPMC 0.2%. * Statistically significant difference with respect to the negative control (*p* < 0.05).

With regard to the in vitro tolerance of formulations with active substances (Figure 2), the hTERT-HCECs cells were exposed to the formulation during 1 or 4 h, stabilizing 0.9% NaCl (negative control) as 100% cell viability. 

The formulation FL-B showed a 93.30 ± 6.63% cell viability after 1 h exposure and 95.09 ± 15.33% after 4 h. In addition, when polymer was incorporated into the formulation (FLP-B), the viability values increased to 107.01 ± 8.74% and 105.33 ± 12.66% at 1 h and 4 h, respectively, however the difference was not statistically significant (*p* > 0.05). On the contrary, CCB showed a viability of 12.44 ± 0.28% after 1 h and 3.18 ± 0.76% after 4 h. The difference between the cell viability after the exposition with the commercial formulation was statistically significantly lower than that of both liposomal formulations at 1 and 4 h (*p* < 0.05 in all cases).

The data of cell viability obtained for the formulations containing travoprost was also higher for the liposomal formulations, all of them being above 80% (FL-T: 91.78 ± 1.63% after 1 h and 83.8 ± 5.31% after 4 h; FLP-T: 99.67 ± 8.06% after 1 h and 96.76 ± 11.71% after 4 h) in comparison to the data obtained for the marketed eye-drop (CCT: 87.97 ± 3.90% after 1 h and 51.55 ± 6.47% after 4 h) (Figure 2).

The 1-h exposure showed no significant differences between the CCT and FL-T formulations (*p* = 0.067), although resulted as being significant between the CCT and FL-P formulations (*p* = 0.046). In the 4-h exposure assays, both liposomal formulations showed significant differences compared with the commercial formulation CCT (*p* = 0.003 for FL-T and *p* = 0.004 for FLP-T). Furthermore, as it can be seen, the inclusion of HPMC appeared to produce a slight increase in cell viability, although the difference with the liposomal formulation without HPMC was not significant (*p* > 0.05). 

#### 3.3.2. In Vitro Tolerance Evaluation in Human Conjunctival Cells

**Figure 3:**
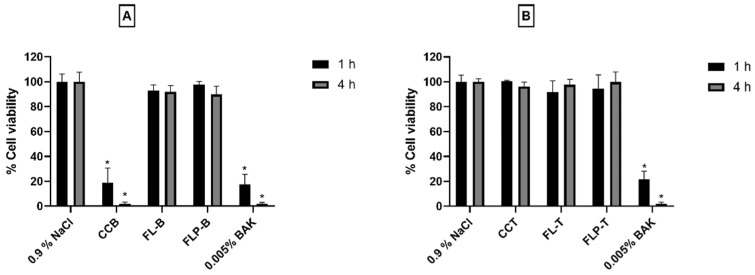
In vitro toxicity evaluation in human conjunctival cells of liposomal formulations containing brimonidine (FL-B and FLP-B) (**A**) or travoprost (FL-T and FLP-T) (**B**) after 1 and 4 h exposure. FL-B: Brimonidine 0.2%, DOPC 7.5 mg/mL, DMPC 2.5 mg/mL, vitamin E 0.1 mg/mL Ubiquinol 0.025 mg/mL, Taurine 0.5%, Ribitol 0.5%; FLP-B: Brimonidine 0.2%, DOPC 7.5 mg/mL, DMPC 2.5 mg/mL, Vitamin E 0.1 mg/mL Ubiquinol 0.025 mg/mL, Taurine 0.5%, Ribitol 0.5%, HPMC 0.2%. FL-T: 40 µg/mL, DOPC 7.5 mg/mL, DMPC 2.5 mg/mL, vitamin E 0.1 mg/mL Ubiquinol 0.025 mg/mL, Taurine 0.5%, Ribitol 0.5%; FLP-T: Travoprost 40 µg/mL, DOPC 7.5 mg/mL, DMPC 2.5 mg/mL, vitamin E 0.1 mg/mL Ubiquinol 0.025 mg/mL, Taurine 0.5%, Ribitol 0.5%, HPMC 0.2%. * Statistically significant difference with respect to the negative control (*p* < 0.05).

After obtaining suitable values of cell viability with liposomal formulations in corneal cells, these were also tested on human conjunctival cells (IM-HConEpiC) (Figure 3). As in the in vitro tolerance tests on corneal cells, 0.9% NaCl was used as a negative control. The resulting cell viability upon exposure of the FL-B and FLP-B formulations for 1 h was above 90% in both formulations, being 93.02 ± 4.53% for FL-B and 97.61 ± 2.59% in the case of FLP-B. On the other hand, cell viability after 4 h of exposure was above 89% for the liposomal formulations with and without polymer (89.8 ± 6.82% and 91.89 ± 5.04%, respectively).

Under the same experimental conditions, a cell viability value of 91.78 ± 9.02% was obtained for the FL-T formulation and 94.37 ± 11.13% for FLP-T after 1 h of exposure. Furthermore, when the exposure was 4 h, cell viability did not decrease for either formulation (97.67 ± 4.41% and 99.88 ± 8.23%, respectively). Thus, in all cases, cell viability remained above 90%, exceeding 95% with an exposure of 4 h (Figure 3). No statistically significant differences were observed between the liposomal formulations of either brimonidine or travoprost (*p* > 0.05). There was also no difference between the commercial formulation of travoprost (CCT) and the liposomal formulations or the negative control. However, the commercial formulation of brimonidine (CCB) produced a statistically significant decrease in cell viability at both 1 and 4 h (*p* < 0.05).

#### 3.3.3. Osmoprotection Studies

**Figure 4:**
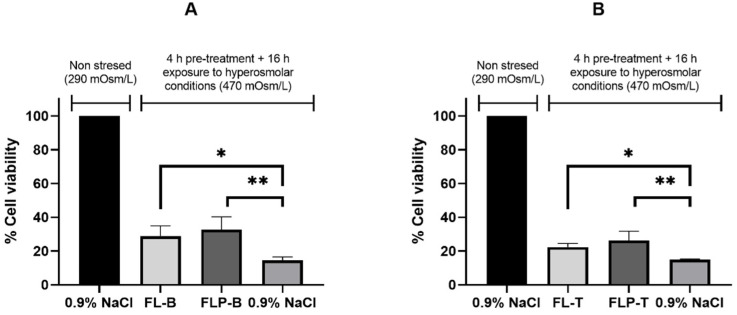
Osmoprotective effect after liposomes pre-incubation during 4 h and exposure to a 470 mOsm/L NaCl solution during 16 h. Evaluation of liposomal formulations containing brimonidine (**A**) or travoprost (**B**) in hTERT-HCECs cells (* *p* < 0.05; ** *p* < 0.005).

For osmoprotection studies, a hyperosmolar stress model was used to detect the osmoprotective activity of different substances. To this, a pre-treatment with the formulations was performed for 4 h, and then the cells were subjected to a chronic 16-h hyperosmolar stress. When the cells were pre-exposed to the liposomal formulations with and without polymer, there was a statistically significant (*p* < 0.05) increase in cell viability for all formulations after the hyperosmolar stress induction compared to the positive control (pre-treatment with 0.9% NaCl) (Figure 4). 

Viability data after hyperosmolar stress in the assay performed to evaluate the brimonidine formulations were as follows: FL-B (28.96 ± 6.02%), FLP-B (32.60 ± 7.75%), and NaCl 0.9% (16.38 ± 4.93%). The liposomal formulation produced a significant increase in cell viability compared to the positive control (*p* < 0.05). Moreover, when HPMC was added to the formulation, the increase in cell viability resulted as being higher and being statistically significant compared to the positive control (*p* < 0.005), although there was no significant difference between formulations. These results mean an increase in the cell viability after hyperosmolar stress of 12.6% and 16.2% when the cells were pre-exposed to FL-B and FLP-B, respectively.

In the case of formulations, including travoprost, the viability results in stressed cells were the following: FL-T (22.40 ± 2.08%), FLP-T (26.27 ± 5.52%), and NaCl 0.9% (14.87 ± 0.47%). Liposomal formulations promoted a significant increase (*p* < 0.05 and *p* < 0.005 for FL-T and FLP-T, respectively) of cell viability compared to NaCl in the hypertonic stress conditions. Accordingly, both formulations showed superior osmoprotective activity with the addition of HPMC. The increase in cell viability after hyperosmolar stress was 7.55% and 11.4% when the cells were pre-exposed to FL-T and FLP-T, respectively.

### 3.4. In Vivo Hypotensive Efficacy Studies 

#### 3.4.1. Brimonidine Liposomal Formulations

The potential reduction in intraocular pressure observed in rabbits receiving the liposomal formulations was evaluated and compared to that produced by the marketed reference formulations. Previously, it was shown that unloaded liposomal formulations, without and with HPMC (FL and FLP), had no effect on the intraocular pressure of the animals since, upon instillation, they produced intraocular pressure values statistically similar (*p* > 0.05) to the values obtained before installation and considered as 100% IOP.

**Figure 5:**
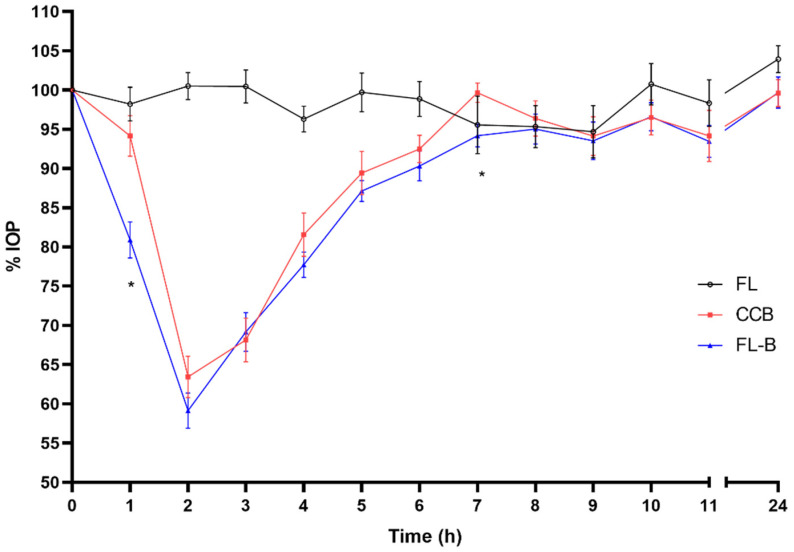
Decrease in intraocular pressure after administration of the liposomal formulation containing brimonidine (0–24 h), with statistical significance between CCB and FL-B formulations (* *p* < 0.05).

**Figure 6:**
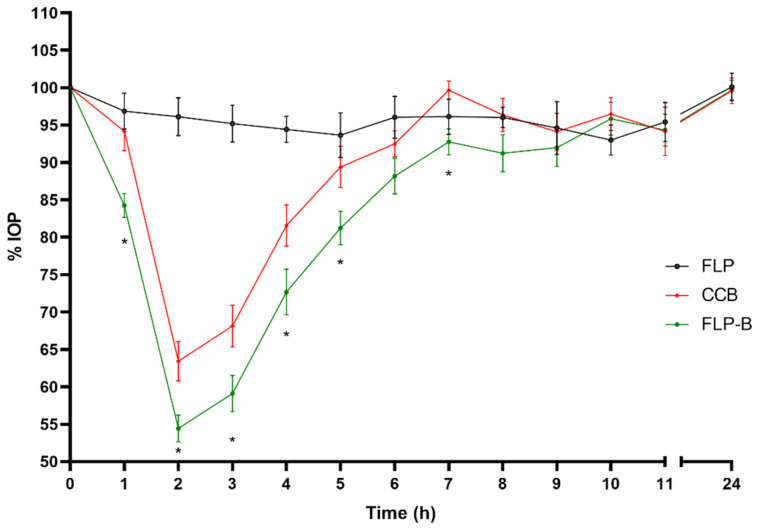
Decrease in intraocular pressure after administration of the liposomal formulation containing brimonidine and HPMC (0–24 h), with statistical significance between CCB and FLP-B formulations (* *p* < 0.05).

**Figure 7:**
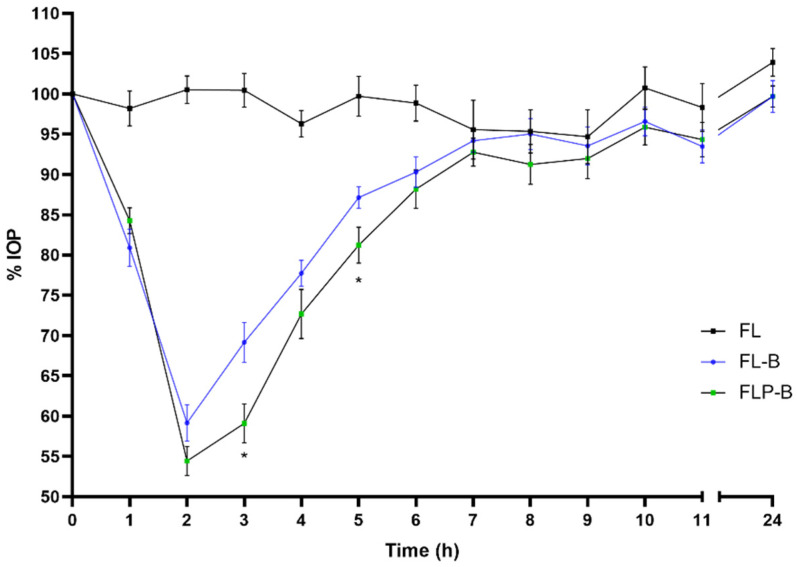
Decrease in intraocular pressure after administration of the liposomal formulation containing brimonidine and HPMC (0–24 h), with statistical significance between FL-B and FLP-B formulations (* *p* < 0.05).

As shown in Figure 5, when comparing the commercial with the FL-B formulation, a maximal reduction in intraocular pressure 2 h after instillation was observed in both cases, however, the hypotensive effect onset occurred faster for FL-B (1h for the liposomal formulation and at 2 h for the marketed one). This was corroborated by the statistical treatment, so one hour after administration of the brimonidine commercial preparation (CCB) the intraocular pressure remained at 94.15 ± 8.21% compared to the IOP at baseline (100%), not being statistically different (*p* = 0.051, CI95 (88.28–100.02)), and the Onset time (t onset) was not until 2 h of administration (*p* < 0.0001, CI95 (57.48–69.38)). In contrast, liposomal formulation FL-B showed an IOP of 80.90 ± 7.29% 1 h after the administration compared to baseline, which means a significant decrease (*p* < 0.0001, CI95 (75.69–86.12)). The inclusion of HPMC in the formulation (FLP-B) kept this similar initial behavior of the liposome formulation (Figure 6 and Figure 7), also showing an onset of action one hour after instillation, with a reduction of the intraocular pressure to 84.26 ± 5.05% (*p* < 0.0001, CI95 (80.65–87.87)). Furthermore, the maximum effect after was also observed after two hours of administration. 

Regarding the maximum IOP reductions (ΔIOP_max_), the results (mean ± SD) were 36.57 ± 8.33, 40.85 ± 7.11, and 45.57 ± 5.05 for CCB, FL-B, and FLP-B formulations, respectively, showing significant differences between CCB and FLP-B (*p* = 0.009), demonstrating a beneficial effect on the IOP reduction by combining the use of liposomes and HPMC.

**Figure 8:**
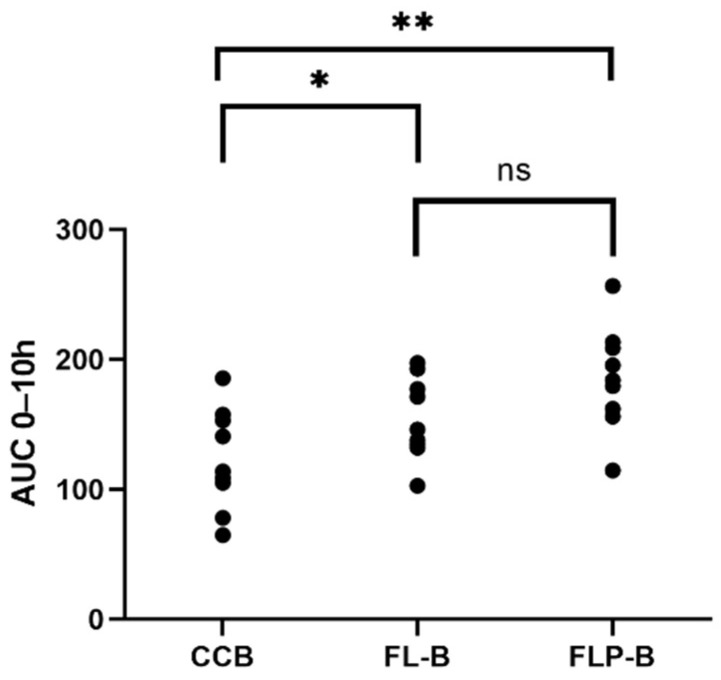
Area under curve values from time 0 h to time 10 h obtained after the instillation of the different formulations, representing the value of each eye. * *p* < 0.05, ** *p* < 0.01, ^ns^ Non Significant.

The formulation of brimonidine in liposomes provoked an increment in the hypotensive effect time period. Seven hours post-instillation, IOP values produced by the commercial formulation CCB (99.66 ± 3.92%) were no longer significant with respect to 100% IOP (*p* = 0.79; CI95 (96.86–102.46)). On the contrary, 9 h after the administration, the liposomal formulations FL-B and FL-P still showed an IOP of 93.53 ± 7.55% (*p* = 0.02; CI95 (88.13–98.93)) and 91.99 ± 7.92% (*p* = 0.02; CI95 (86.32–97.66)), respectively, which means a statistically significant prolongation of the effect. At 10 h, the differences were no longer significant for FL-B and FLP-B. Based on these results, it was decided to analyze the area under the curve between time 0 and time 10 h (AUC_0–10h_) (Figure 8). The AUC_0–10h_ results expressed in mean ± standard deviation (SD) were as follows: CCB: 122.44 ± 37.26; FL-B: 154.62 ± 29.48, and FLP-B: 186.19 ± 38.03.

Subsequently, a determination was made of the differences between the AUC_0–10h_ and the ΔIOP_max_ obtained for each formulation (Supplementary material Tables S1–S3). When comparing the AUC_0–10h_ values, it was observed that both FL-B (*p* = 0.045) and FLP-B (*p* = 0.001) showed a significant difference compared to commercial formulation. The ocular bioavailability for FL-B and FLP-B, evaluated as the corresponding AUC_0–10h_, resulted in being 1.3 and 1.5 times higher, respectively, than after instillation of CCB. Between the two liposomal formulations (with and without HPMC), a slight, but not significant (*p* = 0.053), difference was observed.

#### 3.4.2. Travoprost Liposomal Formulations

**Figure 9:**
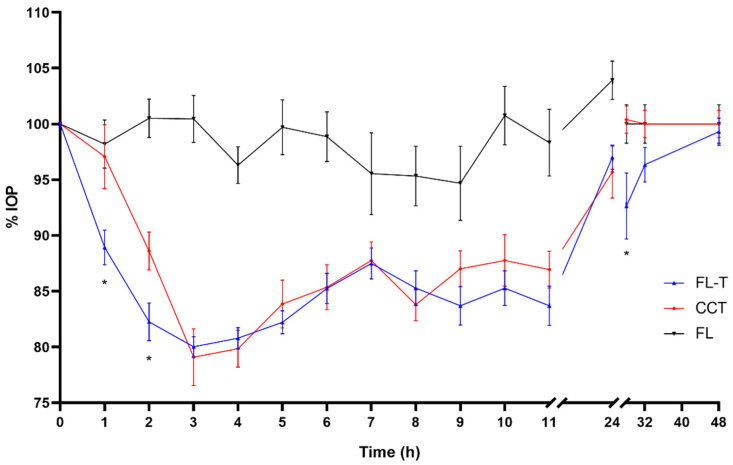
Decrease in intraocular pressure after administration of the liposomal formulation containing travoprost (0–48 h), with statistical significance between CCT and FL-T formulations (* *p* < 0.05).

**Figure 10:**
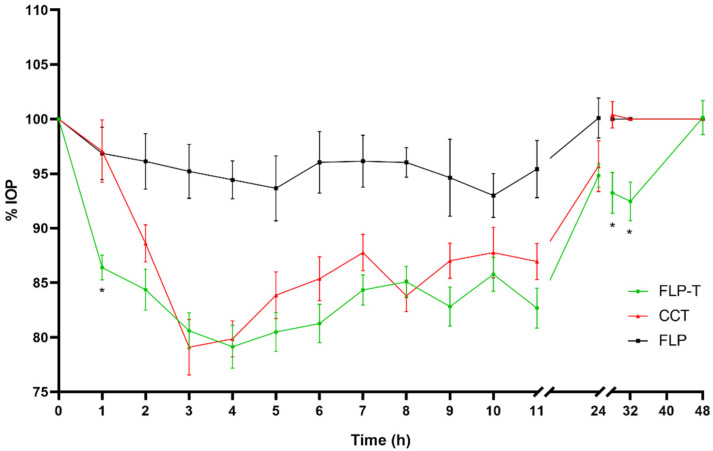
Decrease in intraocular pressure after administration of the liposomal formulation containing travoprost and HPMC (0–48 h), with statistical significance between CCT and FLP-T formulations (* *p* < 0.05).

**Figure 11:**
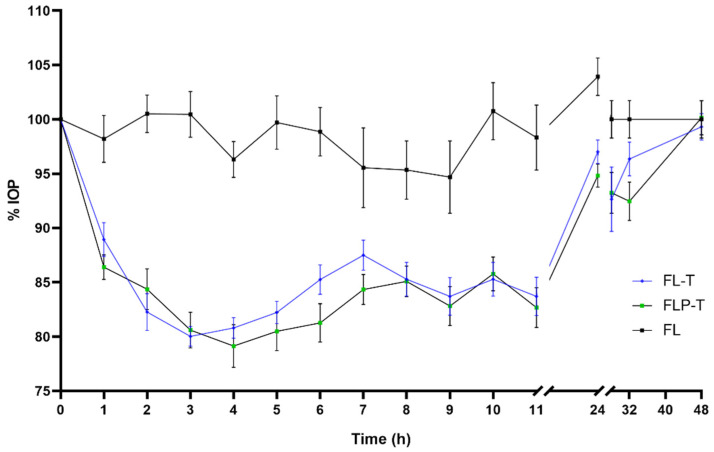
Decrease in intraocular pressure after administration of the liposomal formulation containing travoprost and HPMC (0–48 h), with statistical significance between FL-T and FLP-T formulations.

The effect in IOP reduction was more extended but less pronounced using travoprost formulations. Efficacy studies of the hypotensive effect of the formulations prepared with travoprost could be extended up to 48 h after the single administration (Figure 9, Figure 10 and Figure 11). At neither of these long times was any hypotensive effect of the unloaded liposomal formulations (FL and FLP) observed (*p* > 0.05).

Differences were observed between the commercial formulation and the liposomal formulations (FL-T and FLP-T) loaded both with travoprost, showing a clear improvement in efficacy over CCT in several aspects. Firstly, the onset time of the hypotensive effect was shorter for the liposomal formulations. As can be seen in Figure 10 and Figure 11, 1 h after CCT administration the IOP was still 97.07 ± 9.08%, which was not statistically significant (*p* = 0.334, CI95 (90.57–103.57)) compared to the initial IOP (100%). It was not until 2 h after administration when the IOP significantly decreased to 88.61 ± 5.36% (*p* < 0.0001, CI95 (84.78–92.44)) compared to baseline. By contrast, administering the liposomal formulations the IOP decreased significantly in only 1 h after administration in both cases, being 88.93 ± 4.92 (*p* < 0.0001, CI95 (85.41–92.45)) and 86.39 ± 3.60 (*p* < 0.0001, CI95 (83.81–88.97)) for FL-T and FLP-T, respectively. 

Secondly, significant improvements were also observed in the duration of IOP reduction. When rabbits were treated with the commercial formulation, 24 h after administration the IOP still showed a reduction of 94.31 ± 7.39% compared to baseline, however this was not significant (*p* = 0.098, CI95 90.40–100.97)). In contrast, liposomal formulations FL-T and FLP-T still both showed a statistically significant reduction 32 h after administration: The IOP remained at 96.35 ± 4.90% (*p* = 0.043, CI95 (92.88–99.85)) and 92.46 ± 5.57% (*p* = 0.002, CI95 (88.47–96.44)), respectively. No significant differences were observed for any formulation at 48 h after administration.

**Figure 12:**
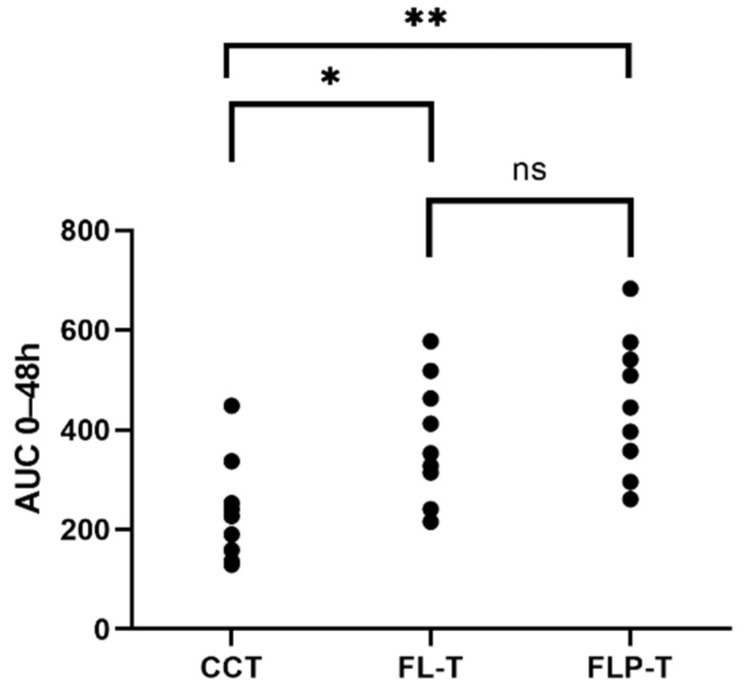
Area under curve from 0 to 48 h of the different formulations, representing the value of each eye. * *p* < 0.05, ** *p* < 0.01, ^ns^ Non Significant.

Furthermore, an improvement in ocular bioavailability was also observed, calculated as the areas under the curve of IOP reduction effect between time 0 and 48 h (AUC_0–48h_) (Figure 12). The AUC_0–48h_ were as follows (mean ± SD): 258.54 ± 117.99 for CCT; 388.58 ± 117.66 for FL-T; 442.69 ± 133.20 for FLP-T. Subsequently, a determination was made of the differences between the AUC_0–48h_ (Supplementary material Tables S4–S6), the CCT data being statistically lower than the liposomal formulations (*p* = 0.023 and *p* = 0.004 for FL-T and FLP-T, respectively). However, the difference was not found to be significant when compared the areas of the two liposomal formulations (*p* = 0.348). According to these calculations, it can be said that the ocular bioavailability of FL-T and FLP-T were 1.5 and 1.7 times greater than CCT, respectively.

However, regarding the ΔIOP_max_ (Supplementary material Tables S4–S6), no significant differences were observed between the 3 formulations (CCT: 20.91 ± 8.03; FL-T: 19.97 ± 2.87; FLP-T 20.86 ± 6.19 expressed in mean ± SD). While for the commercial formulation and the FL-T formulation showed a maximum IOP reduction at 3 h, for the FLP-T formulation it was at 4 h. Apparently, this formulation, which contained HPMC, showed a more gradual and prolonged effect.

## 4. Discussion

The potential of liposomes as drug delivery systems for ocular surface is enormous and increasingly evident. They are investigated to treat pathologies of the ocular surface itself, as is the case for numerous ocular surface infections, as well as for anesthesia [9] and to increase ocular bioavailability in the anterior segment of the eye [40]. In this sense, several authors have explored the possibility of combining hypotensive agents with liposomes to reduce intraocular pressure, such as latanoprost [41] or acetazolamide [42] loaded into soy phosphatidylcholine liposomes.

**Figure 13:**
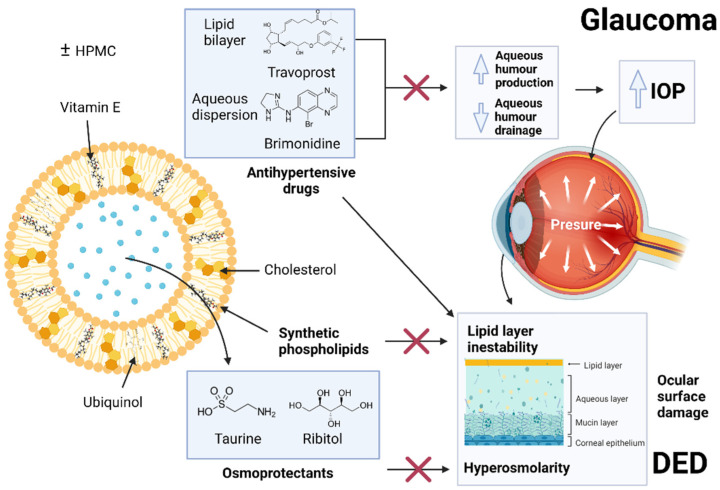
Liposome structure and mechanism for the treatment of glaucoma and prevention of dry eye.

Furthermore, liposomes have been also evaluated for the development of artificial tears to treat dry eye [9], with or without active ingredients [43]. In fact, there are already artificial tears based in liposomes in the market [44]. The aim of our study was to create liposomes loaded with antihypertensive agents included in an osmoprotective vehicle able to control the IOP while preventing the adverse effects of the medication, which can cause damage to the ocular surface [31] as summarized in Figure 13.

The formulations developed included the synthetic phospholipids DOPC and DMPC in their composition. In the literature, numerous authors, including our research group, have used phosphatidylcholine for liposome formation. In addition to being well tolerated [36,45], phosphatidylcholine is one of the major components of the ocular surface [46]. However, despite their advantages, the composition of natural phosphatidylcholines is not exactly known, which can attract reproducibility issues, among others. In this work we decided to use neutral synthetic phospholipids (DOPC and DMPC) with homogenous fatty acid composition for liposome preparation, which have been shown to be well tolerated in topical administration [47]. A great number of studies have been carried out with synthetic phospholipids for the preparation of liposomes showing good stability [48]. Additionally, DOPC has a similar transition temperature to phosphatidylcholine (approximately −16.5 °C) [49], and the addition of DMPC, which has a higher transition temperature [50], can increase the rigidity of liposomes. The antioxidant capacity of DOPC is also remarkable [51]. Previously, liposomes composed of these synthetic phospholipids had been tested by our research group in terms of in vitro tolerance in ocular surface cells with and without antioxidant and osmoprotective compounds [47].

Our liposomal formulations were composed by several components with antioxidant and osmoprotective activity. Antioxidant compounds were included in the lipid bilayer, in addition to cholesterol, this last one providing rigidity to liposomes [52]. Vitamin E is a well-known hydrophobic antioxidant, traditionally included in liposomes to preserve from lipid peroxidation [53]. Additionally, ubiquinol was also included in the formulation, whose antioxidant properties have been already described by several authors [54]. Regarding the additional protection to avoid the development of DED, ribitol, considered a cryoprotectant compound [23,55], as well as osmoprotectant [56], was included in the vehicle. In addition, taurine was also added. Several studies have demonstrated a significant osmoprotective and antioxidant effect in corneal epithelial cells for this aminoacid [24]. In fact, it has already demonstrated an osmoprotective activity in a hyperosmolar model in corneal cells developed by our group [38]. The osmoprotective activity of these substances were also demonstrated in this work using a hyperosmolar model in corneal cells. The use of a borate buffer allows the maintenance of the pH, and also acts as a preservative of the formulation due to its antimicrobial capacity reported by other authors [57].

All the liposomal formulations were designed to offer physicochemical characteristics suitable for administration on the ocular surface (Table 2). In addition, the vesicle size resulted close to 200 nm, which allows for decreased immunogenicity and evasion of phagocytosis uptake [9]. The increase in size observed after HPMC addition could be explained by the coating of the liposomal vesicles with the polymer, a fact previously reported by other authors [4]. The zeta potential was neutral for all the formulations (−10–10 mV). The surface tension for all formulation was below the values observed in tear film for normal individuals and patients with DED (43.6 ± 2.7 mN/m and 49.6 ± 2.2 mN/m respectively), which allows the formulation to be well extended in the ocular surface [46]. Regarding the viscosity, the tear film values fall within 1–8.3 mPa·s, so the values of our formulations (Table 2 and Table 3) were found to be within the range [9]. As previously mentioned, the hypotonicity of the formulations will also counteract the effect of the hypertonicity present in the DED [21].

All the brimonidine initially included for preparation of the formulations was found in the formulation FL-B, while around 11% of travoprost was lost during preparation of liposomal formulation (FL-T). This difference can be explained by the preparation method of the liposomal formulations: Brimonidine was incorporated as an aqueous solution into the lipid layer formed in the round-bottom flask, thus being less subject to losses. However, travoprost is incorporated into the chloroform solution together with other oily components to form the lipid layer. This means that it will be more subject to losses in fabrication, as it must be mechanically incorporated into the aqueous dispersion. Regarding the encapsulation efficiency, for FL-B it was 24.80 ± 0.32%, while for FL-T it was ≥99.01%, which can be easily explained by the nature of the active ingredients. Brimonidine, a water-soluble active ingredient, was distributed into both the aqueous dispersion, and the aqueous core inside the liposomes [58], travoprost, which is liposoluble in nature, was incorporated entirely into the lipid bilayer.

In vitro tolerance studies of the formulations resulted in all liposomal formulations in cell viability values higher than 80% then being over the “good tolerance” limit. This provides us with very promising results, as the studies were conducted on two human cell lines present on the ocular surface: cornea and conjunctiva [59].

In the studies carried out in human corneal hTERT-HCECs cells, the cell viability of our formulations was compared with commercial formulations of both active ingredients (CCB and CCT), because the human corneal line is sensitive enough to show differences in the tolerance. In the case of CCB (Figure 2), cell viabilities were much lower (less than 15% cell viability in only one hour of exposition), probably due to the use of the preservative BAK, which has been shown to be toxic to the ocular surface [31,60]. The same can be observed in conjunctival cells for the CCB formulation (Figure 3), with a significant decrease in cell viability (*p* < 0.05) after only one hour of exposure. The commercial formulation containing travoprost (CCT), despite not containing this preservative, showed a cell viability of approximately 50% after 4 h of exposure in human corneal cells (Figure 2). Therefore, our liposomal formulations seem to be of great advantage in maintaining the integrity of the ocular surface, as cell viability was considerably higher. In addition, cell viability values for formulations with HPMC polymer (FLP-B and FLP-T) outperformed those without HPMC (FL-B and FL-T), with cell viability values higher than 95%. These results agree with previous research, in which HPMC has shown to have protectant activity in corneal and conjunctival cells [38,61]. Considering the extensive literature on the adverse effects of antiglaucomatous formulations [62,63,64], the fact that our formulations show such a high tolerance in cornea and conjunctiva cells would be a great advantage, both to reduce the side effects and to avoid the development of dry eye symptoms.

To complete the tolerance study in ocular surface cells, conjunctival cells were used. When exposed the formulations to conjunctival IM-HConEpiC cells, a minimum of 89% viability was obtained in all conditions for liposomal formulations. Very relevant results were obtained, especially in the case of travoprost formulations (FL-T and FLP-T), where more than 97% viability was achieved after 4 h of exposure. This, together with the promising cytotoxicity results observed in corneal cells, leads us to believe that these formulations could be good candidates for the safe administration of drugs on the ocular surface treatment of ocular pathologies.

The high correlation between topical glaucoma treatment and damage to the ocular surface [65] leading to DED, makes the development of novel formulations able to protect the ocular surface from the damage created in this type of chronic treatment necessary. As it has been widely described in the literature, hyperosmolarity plays a crucial role in DED. Increased tear evaporation or decreased tear production in the DED will increase the osmolarity of the tear film [20]. This increase in osmolarity will trigger inflammatory processes and damage to the corneal epithelium and conjunctiva, which are worsened by the inflammation in a vicious cycle [21], as extensively explained in the introduction.

In vitro osmoprotection studies could help to select excipients and to test formulations as preliminary studies. Our group has developed an in vitro model in corneal cells capable of simulating hyperosmolar stress [38]. In this model, a 16-h exposure to a 470 mOsm/L hyperosmolar solution was used to simulate hyperosmolar stress. This exposure resulted in a decrease in cell viability, as well as a related increase in apoptosis in corneal cells. In addition, by pre-exposure to different substances, this model allows us to sensitively detect the osmoprotection activity produced by different excipients or formulations.

The results demonstrated a significant increase in cell viability of human corneal cells when, prior to hyperosmolar stress (16 h), they were pre-exposed for only 4 h to the proposed liposomal formulations. For all of them the increase in cell viability was statistically significant with respect to the positive control (*p* < 0.05), in which the pre-exposure was carried out with NaCl 0.9% (Figure 4). In both cases (brimonidine and travoprost liposomal formulations), the inclusion of 0.2% HPMC in the vehicle statistically increased the osmoprotective activity (*p* < 0.005 for FLP-B and FLP-T). Therefore, at least in vitro, FLP-B and FLP-T appeared to have superior osmoprotective activity than FL-B and FL-T. These results agree with previous studies conducted by our group, where HPMC was shown to have osmoprotective activity in human corneal cells [38]. HPMC, in addition to being reported to protect ocular surface cells [61], has been shown in previous work to increase the bioavailability of liposome-encapsulated drugs [4]. The significant increase in cell viability with short periods of exposure (only 4 h) before the hyperosmolar stress suggests a high osmoprotectant activity, which would be higher in chronic treatments. In vitro osmoprotective capacity shown by our liposomal formulations (FL-B, FLP-B, FL-T, and FLP-T) is promising, as it could protect the ocular surface from damage caused by the hyperosmolarity of dry eye, preventing its symptoms, while treating the increased IOP associated with glaucoma.

In vivo efficacy studies have been performed to assess the hypotensive effect of two hypotensive agents of different polarity (a hydrophilic compound such as brimonidine or a hydrophobic compound such as travoprost) both included in liposomal formulations with or without HPMC in the vehicle. In addition, the hypotensive activity of two commercial formulations containing these same active agents was also evaluated. The European Agency for the Evaluation of Medicines for Human Use recommended the use of 95 confidence intervals and a *p*-value <0.05 to establish differences between formulations [66]. Consequently, these were our criteria for evaluating the treatments.

Formulations containing brimonidine had a more intense but shorter effect than those containing travoprost, which is due to the different mechanism. This behavior is well known and is due to the different mechanisms of action of each of the active agents [6,67].

Analyzing the effect of the brimonidine-containing formulations, we found that our liposomal formulations had a shorter t onset of effect than the commercial formulation (1 h for FL-B and FLP-B vs. 2 h for CCB). The effect of all formulations showed a maximum effect at 2 h after administration, with the reduction in IOP being approximately 40%. This is consistent with observations by other authors, who found that brimonidine has a rapidly ocular surface penetration, with the greatest effect in the first 5 h [68]. As discussed in Section 3.4.1, the ΔIOP_max_ was higher in the treatment with liposomal formulations than with CCB, being significantly greater in the case of the FLP-B formulation versus CCB (*p* = 0.009). The duration of effect was 7 h for CCB and 10 hs for the liposomal formulations (FL-B and FLP-B) and the ocular bioavailability, calculated as the AUC_0–10h_, was 1.3 and 1.5 times higher for FL-B and FLP-B compared to CCB, respectively, showing in both cases significant differences (*p* < 0.05). Therefore, the inclusion of part of the brimonidine in liposomal vesicles allowed for a faster, greater, and longer effect. It is noteworthy that our liposomal formulations do not contain BAK, unlike the commercial CCB formulation. BAK causes damage to the cornea but also leads to increased penetration of drugs through the cornea [69], so overcoming the effects on IOP produced by the CCB formulation is even more promising, in addition to providing better tolerance in ocular surface cells.

The commercial formulation containing travoprost (CCT) is a novel emulsion composed of aqueous and oily components. However, the adverse effects of this formulation were notable. Indeed, in other works it has been shown that these commercial formulations are cytotoxic in human conjunctival cells [70]. The CCT formulation contains different co-solvents in its composition, which may increase the penetration of the active compound, but also the toxicity of the formulation.

The in vivo efficacy studies of the travoprost formulations proposed in this work showed that the onset time was also faster when travoprost was formulated in liposomes compared to the commercial formulation (CTT). In this case it was also 1 h for FL-T and FLP-T and 2 h for CCT. On the other hand, the duration of effect was much longer (48 h) for FL-T and FLP-T formulations, which outperformed the 28 h of effect observed with CCT. If we focus on the AUC_0–48h_ of the IOP reduction effect as measurement of the ocular bioavailability [71,72], we found that for the liposomal formulations FL-T and FLP-T it was 1.5 and 1.7 times higher than for CCT, respectively. Moreover, the differences between the AUC_0–48h_ of CCT vs. FL-T (Table S5) and CCT vs. FLP-T (Table S6) were statistically significant (*p* < 0.05). As discussed above, although a higher increase in the AUC_0–48h_ for FLP-T was observed, no significant differences were found between the two liposomal formulations. The longer duration of effect of these travoprost-containing liposomal formulations compared to the commercial formulation could be due to the liposoluble nature of travoprost. In fact, the encapsulation efficiency of travoprost was ≥99.01%, meaning that virtually all the active substance was in the lipid bilayer, which could increase internalization by the cells, improving the effect compared to commercial formulation CCT [12]. Although the hypotensive effect did not increase dramatically, the improvement of the effect, together with the remarkably better in vitro tolerance than the commercial formulations in ocular surface cells and their osmoprotective capacity, show the promise of liposomal formulations. The superiority of FLP-B and FLP-T vs. FL-B and FL-T in IOP reduction is consistent with the observations of other authors, who reported improved bioavailability with the use of HMPC in their formulations [4,73]. Additionally, as discussed above, the use of HPMC has been described as protective for the ocular surface, which is consistent with the higher osmoprotective activity of these two formulations discussed previously. Therefore, FLP-B and FLP-T appear to have major advantages for the treatment of glaucoma and the improvement of DED symptoms related with topical antihypertensive treatment.

## 5. Conclusions

The liposomal formulations loaded with both active ingredients (brimonidine and travoprost) developed in our study showed suitable physiochemical characteristics for administration onto the ocular surface, an excellent in vitro tolerance, significantly superior to commercial formulations, osmoprotective properties in a hyperosmolar stress model in human corneal cells, and faster and longer in vivo hypotensive efficacy in normotensive New Zeland albino rabbits compared to commercial formulations. Therefore, liposomal formulations were good candidates for glaucoma treatment while providing ocular surface protection, especially the HPMC-containing formulations (FLP-B and FLP-T), which showed a greater osmoprotective effect on corneal cells, as well as slight improvements in the hypotensive effect compared to those without polymer.

## Figures and Tables

**Table 1:** Composition of the different liposomal formulations prepared.

Formulation	Lipid Bilayer	Aqueous Dispersion
FL-B	DOPC:DMPC 10 mg/mL Cholesterol 1.25 mg/mL Vitamin E 0.1 mg/mL Ubiquinol 0.025 mg/mL	H_3_BO_3_ 8.38% Na_2_B_4_O_7_ 0.755% Ribitol 0.5% Taurine 0.5% Brimonidine 2 mg/mL
FLP-B	DOPC:DMPC 10 mg/mL Cholesterol 1.25 mg/mL Vitamin E 0.1 mg/mL Ubiquinol 0.025 mg/mL	H_3_BO_3_ 8.38% Na_2_B_4_O_7_ 0.755% Ribitol 0.5% Taurine 0.5%, HPMC 0.2%, Brimonidine 2 mg/mL
FL-T	DOPC:DMPC 10 mg/mL Cholesterol 1.25 mg/mL Vitamin E 0.1 mg/mL Ubiquinol 0.025 mg/mL 40 µg/mL Travoprost	H_3_BO_3_ 8.38% Na_2_B_4_O_7_ 0.755% Ribitol 0.5% Taurine 0.5%
FLP-T	DOPC:DMPC 10 mg/mL Cholesterol 1.25 mg/mL Vitamin E 0.1 mg/mL Ubiquinol 0.025 mg/mL 40 µg/mL Travoprost	H_3_BO_3_ 8.38% Na_2_B_4_O_7_ 0.755% Ribitol 0.5% Taurine 0.5% HPMC 0.2%

**Table 2:** Physicochemical characterization of the liposomal formulation with and without HPMC 0.2% containing brimonidine or travoprost. Data are represented as the mean ± the standard deviation of the 3 values.

	FL-B	FLP-B	FL-T	FLP-T
Size (nm)	186.77 ± 65.97	212.33 ± 77.57	156.63 ± 61.83	184.27 ± 73.80
PDI	0.13	0.13	0.16	0.16
Zeta potential (mV)	2.76 ± 0.04	2.66 ± 0.22	0.63 ± 0.06	0.66 ± 0.07
pH	6.55 ± 0.00	6.53 ± 0.01	6.98 ± 0.02	7.03 ± 0.01
Osmolarity (mOsm/L)	226.33 ± 1.15	230 ± 1.00	221.6 ± 0.58	221 ± 1.00
Surface tension (mN/m)	28.37 ± 0.67	27.53 ± 1.01	25.53 ± 0.80	26.13 ± 0.15
Viscosity (mPa·s)	1.05 ± 0.01	3.90 ± 0.02	1.10 ± 0.01	3.93 ± 0.05

FL: DOPC 7.5 mg/mL, DMPC 2.5 mg/mL, vitamin E 0.1 mg/mL, Ubiquinol 0.025 mg/mL, Taurine 0.5%, Ribitol 0.5%; FLP: DOPC 7.5 mg/mL, DMPC 2.5 mg/mL, vitamin E 0.1 mg/mL Ubiquinol 0.025 mg/mL, Taurine 0.5%, Ribitol 0.5%, HPMC 0.2%.

**Table 3:** Quantification and encapsulation efficiency of the active ingredients brimonidine and travoprost in liposomal formulations, determined by HPLC.

	Yield (%)	EE (%)
FL-B (Brimonidine)	100.10 ± 0.34	24.78 ± 0.32
FL-T (Travoprost)	89.15 ± 0.49	≥99.01

## Data Availability

The data presented in this study are available on request from the corresponding author.

## Supplementary Materials

The following supporting information can be downloaded at: https://www.mdpi.com/article/10.3390/pharmaceutics14071405/s1, Table S1: Relationship between the two mean difference test (*p*-value of Student’s test) and confidence intervals for the difference of the means. CCL and FL-B; Table S2: Relationship between the two mean difference test (*p*-value of Student’s test) and confidence intervals for the difference of the means. CCL and FLP-B; Table S3: Relationship between the two mean difference test (*p*-value of Student’s test) and confidence intervals for the difference of the means. FL-B and FLP-B; Table S4: Relationship between the two mean difference test (*p*-value of Student’s test) and confidence intervals for the difference of the means. CCT and FL-T; Table S5: Relationship between the two mean difference test (*p*-value of Student’s test) and confidence intervals for the difference of the means. CCT and FLP-T; Table S6: Relationship between the two mean difference test (*p*-value of Student’s test) and confidence intervals for the difference of the means. FL-T and FLP-T.
