# Supplementary material for: Novel Osmoprotective DOPC-DMPC Liposomes Loaded with Antihypertensive Drugs as Potential Strategy for Glaucoma Treatment
Source: Pharmaceutics. 2022 Jul 4;14(7):1405. doi: 10.3390/pharmaceutics14071405 (PMC9317418; doi:10.3390/pharmaceutics14071405)
Supplement: Supplementary file 1 [file pharmaceutics-14-01405-s001.zip › pharmaceutics-1773291-supplementary.pdf]

Table S1. Relationship between the two mean difference test (P-value of Student's test) and confidence intervals for the difference of the means. CCL and FL-B.

	P-Value for H0: Difference = 0	95% Confidence Interval
Maximal IOP reduction (%)	0.232	(-3.00< FL-B – CCB <11.56)
$AUC_{0\text{–}10h}$ (%·h)	0.045	(0.65 < FL-B – CCB < 63.78)

Table S2. Relationship between the two mean difference test (P-value of Student's test) and confidence intervals for the difference of the means. CCL and FLP-B.

	P-Value for H0: Difference = 0	95% Confidence Interval
Maximal IOP reduction (%)	0.009	(2.52< FLP-B – CCB < 15.47)
$AUC_{0\text{–}10h}$ (%·h)	<0.001	(18.37 < FLP-B – CCB < 99.12)

Table S3. Relationship between the two mean difference test (P-value of Student's test) and confidence intervals for the difference of the means. FL-B and FLP-B.

	P-Value for H0: Difference = 0	95% Confidence Interval
Maximal IOP reduction (%)	0.104	(-10.51 < FLP-B– FL-B <1.07)
$AUC_{0\text{–}10h}$ (%·h)	0.053	(-0.39 < FLP-B– FL-B < 63.55)

Table S4. Relationship between the two mean difference test (P-value of Student's test) and confidence intervals for the difference of the means. CCT and FL-T.

	P-Value for H0: Difference = 0	95% Confidence Interval
Maximal IOP reduction (%)	0.889	(-14.96< FL-T – CCT < 13.08)
$AUC_{0\text{–}48h}$ (%·h)	0.023	(19.33 < FL-T – CCT <240.74)

Table S5. Relationship between the two mean difference test (P-value of Student's test) and confidence intervals for the difference of the means. CCT and FLP-T.

	P-Value for H0: Difference = 0	95% Confidence Interval
Maximal IOP reduction (%)	0.988	(-6.79< FLP-T – CCT < 6.68)
$AUC_{0\text{–}48h}$ (%·h)	0.004	(65.93< FLP-T – CCT <302.37)

Table S6. Relationship between the two mean difference test (P-value of Student's test) and confidence intervals for the difference of the means. FL-T and FLP-T.

	P-Value for H0: Difference = 0	95% Confidence Interval
Maximal IOP reduction (%)	0.685	(-3.66< FLP-T – FL-T < 5.42)
$AUC_{0–48h}$ (%·h)	0.348	(-63.97< FLP-T – FL-T <172.18)
